# Platelet-derived growth factor (PDGF)-BB regulates the airway tone via activation of MAP2K, thromboxane, actin polymerisation and Ca^2+^-sensitisation

**DOI:** 10.1186/s12931-022-02101-x

**Published:** 2022-07-15

**Authors:** Annette D. Rieg, Said Suleiman, Carolin Anker, Nina A. Bünting, Eva Verjans, Jan Spillner, Sebastian Kalverkamp, Saskia von Stillfried, Till Braunschweig, Stefan Uhlig, Christian Martin

**Affiliations:** 1 Department of Anaesthesiology, Medical Faculty RWTH-Aachen, Aachen, Germany; 2 Institute of Pharmacology and Toxicology, Medical Faculty RWTH-Aachen, Aachen, Germany; 3 Department of Paediatrics, Medical Faculty RWTH-Aachen, Aachen, Germany; 4 Department of Cardiac and Thorax Surgery, Medical Faculty RWTH-Aachen, Aachen, Germany; 5 Institute of Pathology, Medical Faculty RWTH-Aachen, Aachen, Germany

## Abstract

**Background:**

PDGFR-inhibition by the tyrosine kinase inhibitor (TKI) nintedanib attenuates the progress of idiopathic pulmonary fibrosis (IPF). However, the effects of PDGF-BB on the airway tone are almost unknown. We studied this issue and the mechanisms beyond, using isolated perfused lungs (IPL) of guinea pigs (GPs) and precision-cut lung slices (PCLS) of GPs and humans.

**Methods:**

IPL: PDGF-BB was perfused after or without pre-treatment with the TKI imatinib (perfused/nebulised) and its effects on the tidal volume (TV), the dynamic compliance (Cdyn) and the resistance were studied. PCLS (GP): The bronchoconstrictive effects of PDGF-BB and the mechanisms beyond were evaluated. PCLS (human): The bronchoconstrictive effects of PDGF-BB and the bronchorelaxant effects of imatinib were studied. All changes of the airway tone were measured by videomicroscopy and indicated as changes of the initial airway area.

**Results:**

PCLS (GP/human): PDGF-BB lead to a contraction of airways. IPL: PDGF-BB decreased TV and Cdyn, whereas the resistance did not increase significantly. In both models, inhibition of PDGFR-(β) (imatinib/SU6668) prevented the bronchoconstrictive effect of PDGF-BB. The mechanisms beyond PDGF-BB-induced bronchoconstriction include activation of MAP2K and TP-receptors, actin polymerisation and Ca^2+^-sensitisation, whereas the increase of Ca^2+^ itself and the activation of EP_1–4_-receptors were not of relevance. In addition, imatinib relaxed pre-constricted human airways.

**Conclusions:**

PDGFR regulates the airway tone. In PCLS from GPs, this regulatory mechanism depends on the β-subunit. Hence, PDGFR-inhibition may not only represent a target to improve chronic airway disease such as IPF, but may also provide acute bronchodilation in asthma. Since asthma therapy uses topical application. This is even more relevant, as nebulisation of imatinib also appears to be effective.

## Background

Platelet-derived growth factor (PDGF)-BB and its receptor PDGFR are strongly involved in the pathogenesis of chronic airway disease [1], as both highly promote proliferation in airways [2]. This instance provides for the evidence that PDGFR-inhibition by tyrosine kinase inhibitors (TKIs) appear to be beneficial in chronic airway disease, e.g. idiopathic pulmonary fibrosis (IPF) [3–5] or asthma [6, 7]. Beyond the involvement of PDGF-BB and PDGFR in proliferation of airways and pulmonary vessels [1, 2, 8], PDGF-BB and PDGFR appear to regulate the tone of airways [9, 10].

The receptor tyrosine kinase PDGFR comprises of the two subunits αα, αβ or ββ which are activated by different ligands, e.g., in vivo PDGFR-α is activated by PDGF-AA and PDGF-CC, whereas PDGFR-β is activated by PDGF-BB [2]. In contrast, further possibilities are conceivable in vitro, e.g. the activation of PDGFR-αβ by PDGF-BB [2]. During organogenesis, the various PDGFR-subunits fulfil different functions, e.g., PDGFR-α is involved in the formation of the lungs, the skin, the gonads and the central nervous system, whereas PDGFR-β is responsible for the formation of blood vessel [2]. In respect of proliferative processes, PDGFR-β promotes the remodelling of the pulmonary vascular bed [11], as well as the remodelling in chronic fibrotic lung disease [12, 13] and asthma [14, 15].

This study was designed to evaluate the contractile effects of PDGF-BB on airway parameters in isolated perfused lungs (IPL) of guinea pigs (GPs) [16–19]. Further, we studied the effects of PDGF-BB in precision-cut lung slices (PCLS) of GPs and humans [17–22]. PCLS resembles an ex vivo model which allows to study the tone of pulmonary arteries, pulmonary veins and airways concurrently within their tissue organization excluding the exposure to in vivo factors such as shear stress, vascular filling pressure or thromboembolism [20, 21, 23]. As a major advantage, PCLS allow to compare how pulmonary vessel and airways react to several stimulants within different species [16, 18, 22, 24–26].

With regard to acute and chronic airway diseases, there are multiple open questions concerning the role of PDGF-BB and PDGFR. We addressed the following points: (1) Does PDGF-BB contract GP airways and is this contraction related to PDGFR-β? (2) How are the effects of PDGF-BB on airway parameters? (3) Does PDGF-BB affect airway parameters, if lungs are pre-treated with the TKI imatinib (perfused/inhaled)? (4) Does PDGF-BB contract human airways? (5) Do TKIs exert bronchodilative properties in human airways? (6) What are the mechanisms beyond PDGF-BB-induced contraction?

## Methods

### Lung tissue from GPs and humans

Female Dunkin Hartley GPs (350 ± 50 g) were obtained from Charles River (Sulzfeld, Germany). All animal care and experimental procedures were approved by the Landesamt für Natur, Umwelt und Verbraucherschutz Nordrhein-Westfalen (ID: 84-02.04.2013A146, 8.87-51.05.20.10.245, 50066A4) and strictly performed due the rules of the Directive 2010/63/EU of the European Parliament.

Human PCLS were prepared from patients undergoing thoracic surgery (lobectomy) due to cancer. After pathological inspection, cancer free tissue from a peripheral part of the lung was selected. In functional lung measurements, none of the patients showed relevant signs of chronic airway disease. The study was approved by the ethics committee (EK 61/09) of the Medical Faculty Aachen, Rhenish-Westphalian Technical University (RWTH) Aachen. All patients gave written informed consent.

### Isolated perfused lungs of the GP

**Fig. 1:**
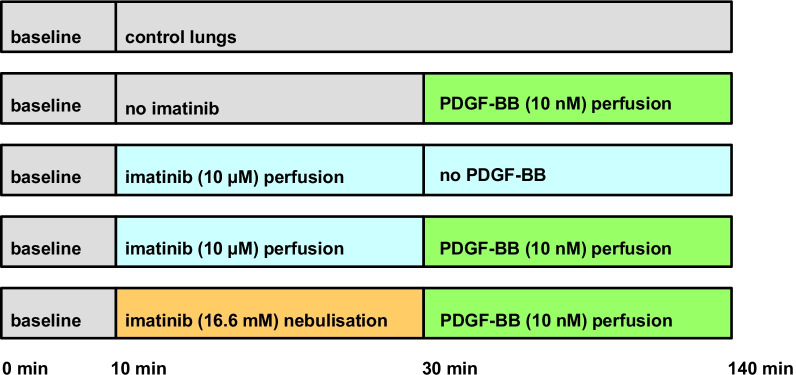
Overview of the timeline. This overview illustrates the different groups and the timeline of all experiments using the IPL

GP lungs were prepared as described previously [17–19, 27]. Briefly, intraperitoneal anaesthesia was performed (pentobarbital: 95 mg/kg) and verified by missing reflexes. The GP was exsanguinated, the trachea cannulated and the lung ventilated with positive pressure with a frequency of 70 breaths/min. Next, the apex of the left ventricle was cut and cannulas were placed in the pulmonary artery (perfusion inflow) and in the left atrium (perfusion outflow). Afterwards, the lung was perfused at constant flow (20 mL/min) with Krebs–Henseleit buffer, containing 2% bovine serum albumin, 0.1% glucose, 0.3% HEPES and 50 nM salbutamol in order to prevent bronchoconstriction [28]. The temperature of the perfusate was maintained at 37 °C with a water bath and the pH was adjusted between limits (7.35 and 7.45) by carbon dioxide. Heart and lungs were withdrawn en-bloc and transferred into a negative-pressure chamber; next, ventilation was switched from positive pressure to negative pressure. To avoid atelectasis of the lung, every 5 min a deep breath was applied. All following parameters were continuously monitored: tidal volume (TV), dynamic compliance (Cdyn), resistance (Res), pulmonal arterial pressure (P_PA_), left atrial pressure (P_LA_) and flow. Once respiratory and haemodynamic parameters remained stable over 10 min (baseline), imatinib was either perfused (10 µM) or nebulised (16.6 mM). Control lungs remained untreated. After 30 min, PDGF-BB (10 nM) was added to the recirculating perfusion buffer (total volume 200 mL) and perfused in untreated and in imatinib-pre-treated lungs. The different groups and the timeline of the experiments are illustrated in Fig. 1.

### Perfusion and nebulisation of imatinib

Using a buffer volume of 200 mL, perfusion of 10 µM imatinib corresponds to a total dose of 1.18 mg imatinib or to 3.5 mg/kg body weight imatinib. To nebulise imatinib, 29.38 mg imatinib mesylate were solved in 3 ml aqua to obtain a solution of 16.6 mM. Afterwards, the solution of imatinib (16.6 mM) was nebulised over 130 min. Supposing a lung flow of 0.21 L/min (70 breaths by 3 mL) and a pressure of 1.5 bar, the total amount of inhaled imatinib corresponds to less than 4% of the nebulised amount of imatinib [29], namely 1.18 mg, corresponding to 3.5 mg/kg body weight imatinib, respectively.

### Precision-cut lung slices (PCLS) from GPs and humans

In GPs, intraperitoneal anaesthesia was performed with 95 mg/kg pentobarbital (Narcoren; Garbsen, Germany) and verified by missing reflexes. The GP was exsanguinated, the trachea cannulated and the diaphragm opened. Thereafter, PCLS were prepared as described before [17–21, 24]. Whole lungs from GP or human lung lobes were filled via the trachea or a main bronchus, respectively with 1.5% low-melting agarose and cooled on ice to harden them. Afterwards, tissue cores (diameter 11 mm) were prepared and cut into 300 µm thick slices with a Krumdieck tissue slicer (Alabama Research & Development, Munford, AL, USA). PCLS were incubated at 37 °C and in order to wash out the agarose, repeated medium changes were performed.

### Identification of the airway, histology

Airways from GPs were identified by their anatomical features; (1) beating cilia indicate the airways including their functional integrity and (2) the airways accompanying the pulmonary arteries [18, 21].

### Pharmacological interventions, measurements and videomicroscopy

To evaluate the contractile effect of PDGF-BB in airways from GPs or humans, PCLS were exposed for 60 min to 100 nM PDGF-BB (Fig. 3A, B). If a signalling pathway was evaluated (Figs. 4, 5, 6 and 7), PCLS were additionally pre-treated for 60 min with one of the following inhibitors at concentrations about 10–100 fold above the IC_50_ value of the target: PDGFR-α: 100 nM ponatinib (IC_50_: 1.1 nM) [30–32]; PDGFR-β: 5 µM SU6668 (IC_50_: 0.008–0.1 µM) [33–35]; PDGFR-α/β: 100 µM imatinib (IC_50_: 0.6–1.8 µM) [36], L-Type Ca^2+^-channels: 100 nM amlodipine (IC_50_: 1.9 nM) [37]; Rho-Kinase: 10 µM fasudile (IC_50_: 1.4 µM) [38]; protein kinase C (PKC): 5 µM calphostin C (IC_50_: 50 nM) [39]; MAP2K: 50 µM PD98059 (IC_50_: 2–7 µM) [40]; MAP2K: 5 µM U0126 (IC_50_: 58–72 nM) [41]; actin polymerisation: 10 µM cytochalasin D (IC_50_: 100 nM) [42]; TP: 10 µM SQ29548 (IC_50_ 10 nM) [43]; IP: 1 µM RO-1138452 (IC_50_: 5–10 nM) [44]; EP_1_: 1 µM SC51322 (IC_50_: 13.8 nM) [45]; EP_2_: 1 µM PF04418948 (IC_50_: 2.7 nM) [46, 47]; EP_3_: 1 µM L798106 (IC_50_: 10 nM) [43, 48] and EP_4_: 1 µM L161982 (IC_50_: 3.2 nM) [43]. To study the relaxing effects of imatinib in human airways (Fig. 3C), human PCLS were incubated with 100 nM Endothelin-1 (ET-1) to induce a stable contraction after 1 h. Subsequently, a concentration–response curve with imatinib was performed. Controls received no further treatment.

In PCLS, all changes of the initial airway area (IAA) were quantified in % and indicated as “Change [% of IAA]”. Thus, an IAA < 100% indicates contraction and an IAA > 100% indicates relaxation. To compare the contractile effect of PDGF-BB in pre-treated airways, the intraluminal area was defined after pre-treatment again as 100% (exceptional Fig. 4). In the graphs, all pre-treatments were indicated. The intraluminal area of airways was monitored with a digital video camera (Leica Viscam 1280, Leica DFC 280). The images were analysed with Optimas 6.5 (Media Cybernetics, Bothell, WA).

### Chemicals

PDGF-BB was provided by Peprotech (Hamburg, Germany). Imatinib mesylate, amlodipine, fasudile, calphostin C, SC51322, PF04418948, L798106 and L161982 were purchased from Tocris Bioscience (Ellisville, Missouri, USA). Ponatinib was acquired from SelleckChem (Munich, Germany). SQ29548, RO-1138452, SU6668, PD98059 and U0126 were acquired from Cayman Europe (via Biomol, Hamburg, Germany). ET-1 was purchased from Biotrends (Wangen, Switzerland). Cytochalasin D or standard laboratory chemicals were provided by Sigma (Steinheim, Germany).

### Statistical analysis

Statistics were conducted using SAS software 9.3 (SAS Institute, Cary, North Carolina, USA) and GraphPad Prism 5.01 (GraphPad, La Jolla, USA). All data, except Fig. 3C were analysed using a linear mixed model analysis (LMM) with the covariance structure AR(1). The data in Fig. 3C were analysed by EC_50_ values (GraphPad Prism). All p-values were adjusted for multiple comparisons by the false discovery rate and are presented as mean ± SEM; n indicates the numbers of animals or human lungs. p < 0.05 is considered as significant.

## Results

We studied the effect of PDGF-BB on the airway tone using the IPL and PCLS from humans and GPs.

### IPL: effect of PDGF-BB on airway parameters

**Fig. 2:**
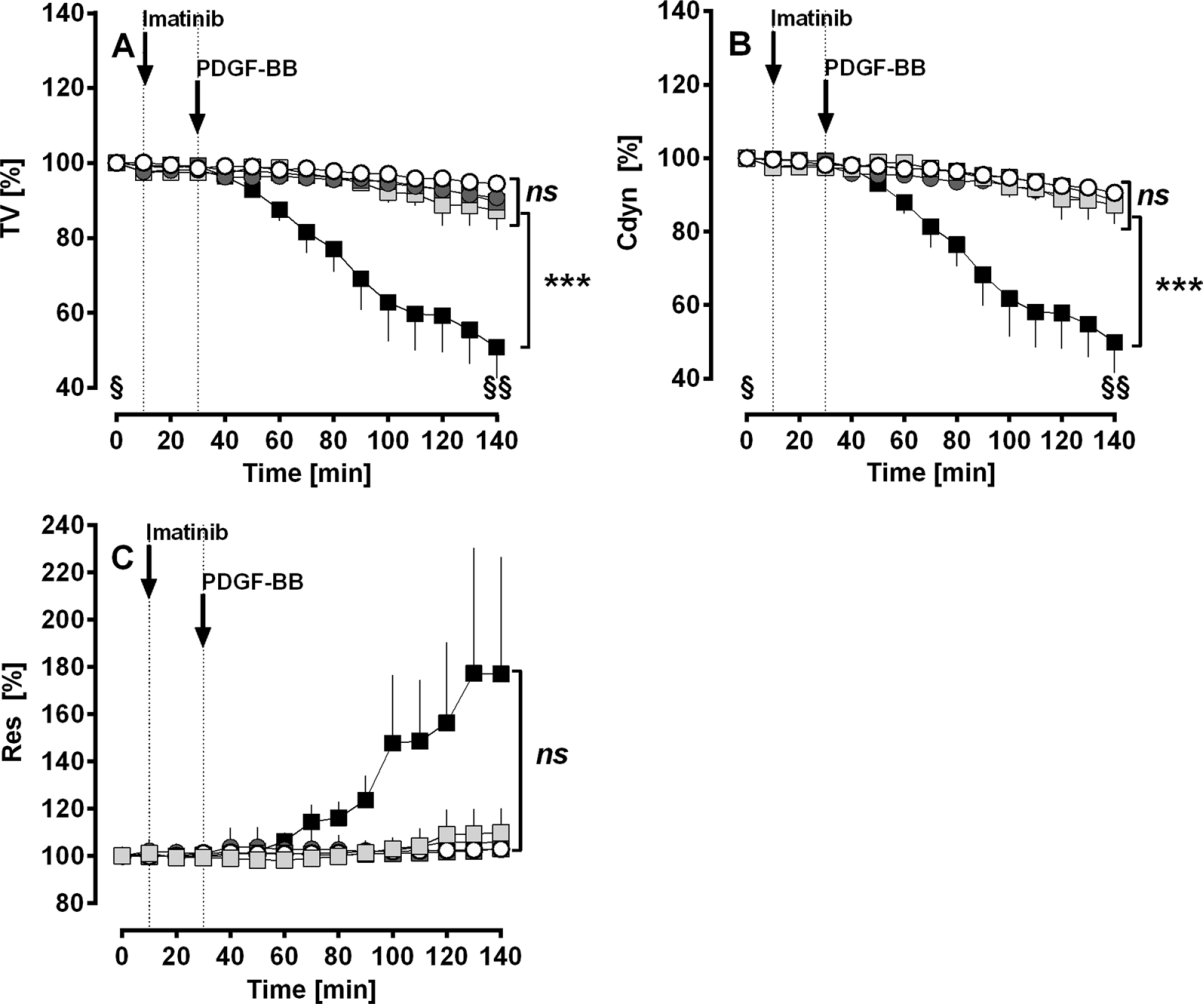
(GPs’ IPL): Effect of PDGF-BB on airway parameters. **A** Effect of PDGF-BB on TV: (○) control (n = 7); (■) PDGF-BB (n = 7); (  ) imatinib (n = 7); (  ) perfused imatinib/PDGF-BB (n = 7); (  ) nebulised imatinib/PDGF-BB (n = 6); ■ PDGF-BB: time point 0 (**§**) vs. 140 (**§§**) min: p < 0.001. **B** Effect of PDGF-BB on Cdyn: (○) control (n = 7); (■) PDGF-BB (n = 7); (  ) imatinib (n = 7); (  ) perfused imatinib/PDGF-BB (n = 7); (  ) nebulised imatinib/PDGF-BB (n = 6); ■ PDGF-BB: time point 0 (**§**) vs. 140 (**§§**) min: p < 0.001. **C** Effect of PDGF-BB on Res: (○) control (n = 7); (■) PDGF-BB (n = 7); (  ) imatinib (n = 7); (  ) perfused imatinib/PDGF-BB (n = 7); (  ) nebulised imatinib/PDGF-BB (n = 6). **A**–**C** Statistics was performed by a LMM. p < 0.05 are considered as significant: *p < 0.05, **p < 0.01 and ***p < 0.001

Perfusion of PDGF-BB decreased the TV (Fig. 2A) and Cdyn (Fig. 2B) up to 50% compared to baseline values and to untreated control lungs (p < 0.001 for all). This effect was completely prevented, if lungs were pre-treated with perfused or nebulised imatinib (Fig. 2A, B). Accordingly, perfusion with PDGF-BB appears to increase Res, but statistical evaluation did not reveal significance (Fig. 2C). Anyhow, pre-treatment with imatinib prevented any changes (Fig. 2C). Imatinib itself had no influence on these airway parameters (Fig. 2A–C).

### PCLS (GP): PDGF-BB contracts airways via activation of PDGFR-β

Next, using PCLS, we tried to find out, if PDGF-BB contracts the airways and if this contraction depends on PDGFR-β, as it was already shown for PVs [17].

**Fig. 3:**
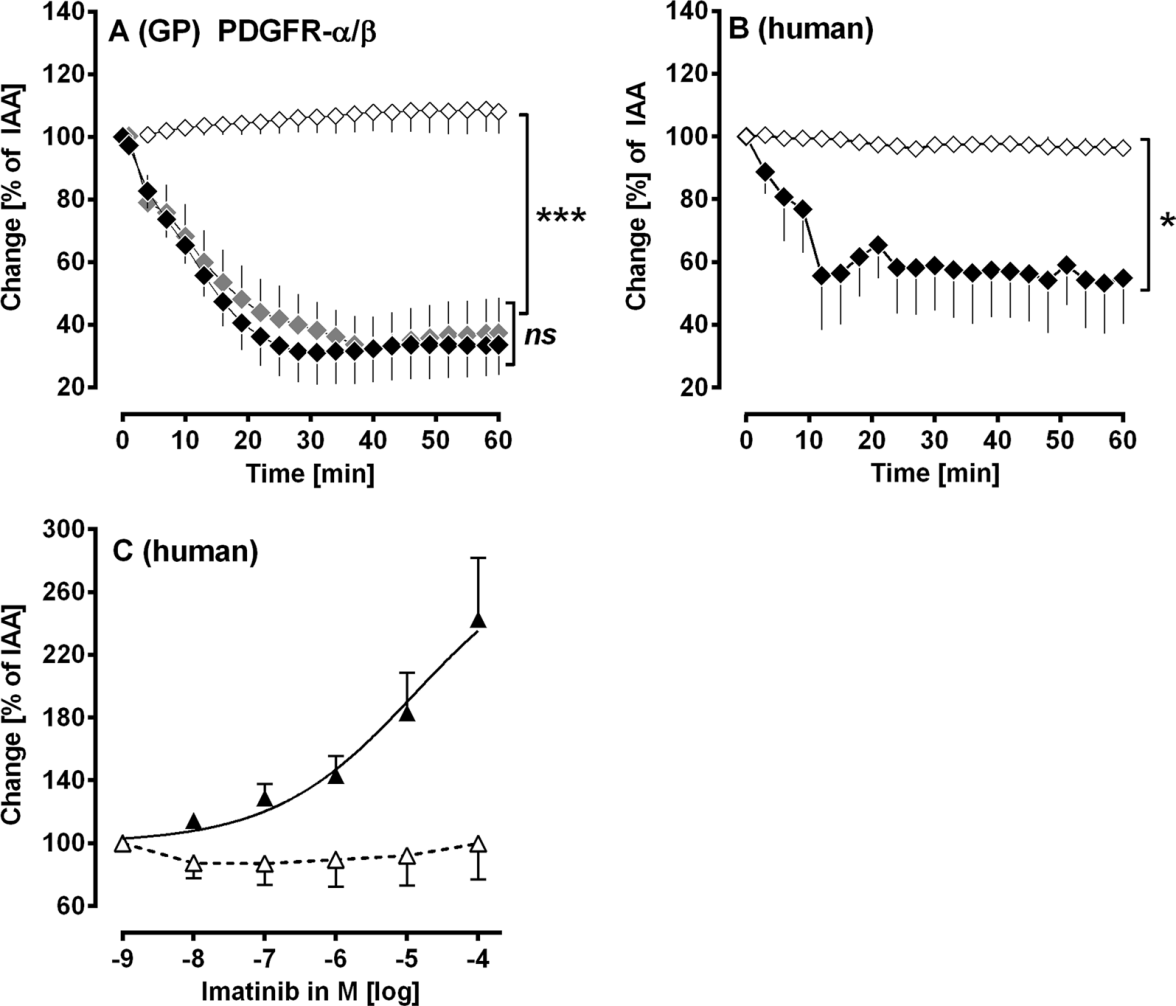
(Human and GPs’ PCLS): PDGF-BB regulates the airway tone via activation of PDGFR-(β). **A** The contractile effect of PDGF-BB in GPs’ airways: (◆) no pre-treatment/100 nM PDGF-BB (n = 7); (  ) pre-treatment with 100 nM ponatinib/100 nM PDGF-BB (n = 7); (◇) pre-treatment with 5 µM SU6668/100 nM PDGF-BB (n = 7). **B** PDGF-BB contracts human airways: (◆) 100 nM PDGF-BB (n = 3); (◇) pre-treatment with 100 µM imatinib/100 nM PDGF-BB (n = 3). **C** Imatinib relaxes human airways: (△) pre-treatment with 100 nM ET-1 (n = 3); (▲) pre-treatment with 100 nM ET-1/imatinib (n = 5). **A**, **B** Statistics was performed by a LMM. **C** Statistics was performed by calculating EC_50_ values by the standard 4-parameter logistic non-linear regression model (GraphPad). p < 0.05 are considered as significant: *p < 0.05, **p < 0.01 and ***p < 0.001

In PCLS, PDGF-BB contracted the airways up to 30% of IAA (p < 0.001) and this effect was prevented, if PCLS were pre-treated with the PDGFR-β-inhibitor SU6668 (Fig. 3A; p < 0.001). In contrast, inhibition of PDGFR-α by ponatinib did not alter PDGF-BB induced bronchoconstriction (Fig. 3A).

### Human PCLS: activation or inhibition of PDGFR-α/β alters the airway tone

PDGF-BB contracted human airways up to 53% of IAA (p < 0.05) and this bronchoconstriction was completely prevented if human airways were pre-treated with the PDGFR-α/β-inhibitor imatinib (Fig. 3B; p < 0.05). Next, we studied if imatinib also relaxes human airways. Therefore, airways were pre-constricted with 100 nM ET-1 prior to the application of increasing concentrations of imatinib (Fig. 3C). Imatinib relaxed human airways strongly up to 260% of IAA (Fig. 3C).

### PCLS (GP): mechanisms for PDGF-BB-induced bronchoconstriction

#### Activation of MAP2K in PDGF-BB-induced bronchoconstriction

**Fig. 4:**
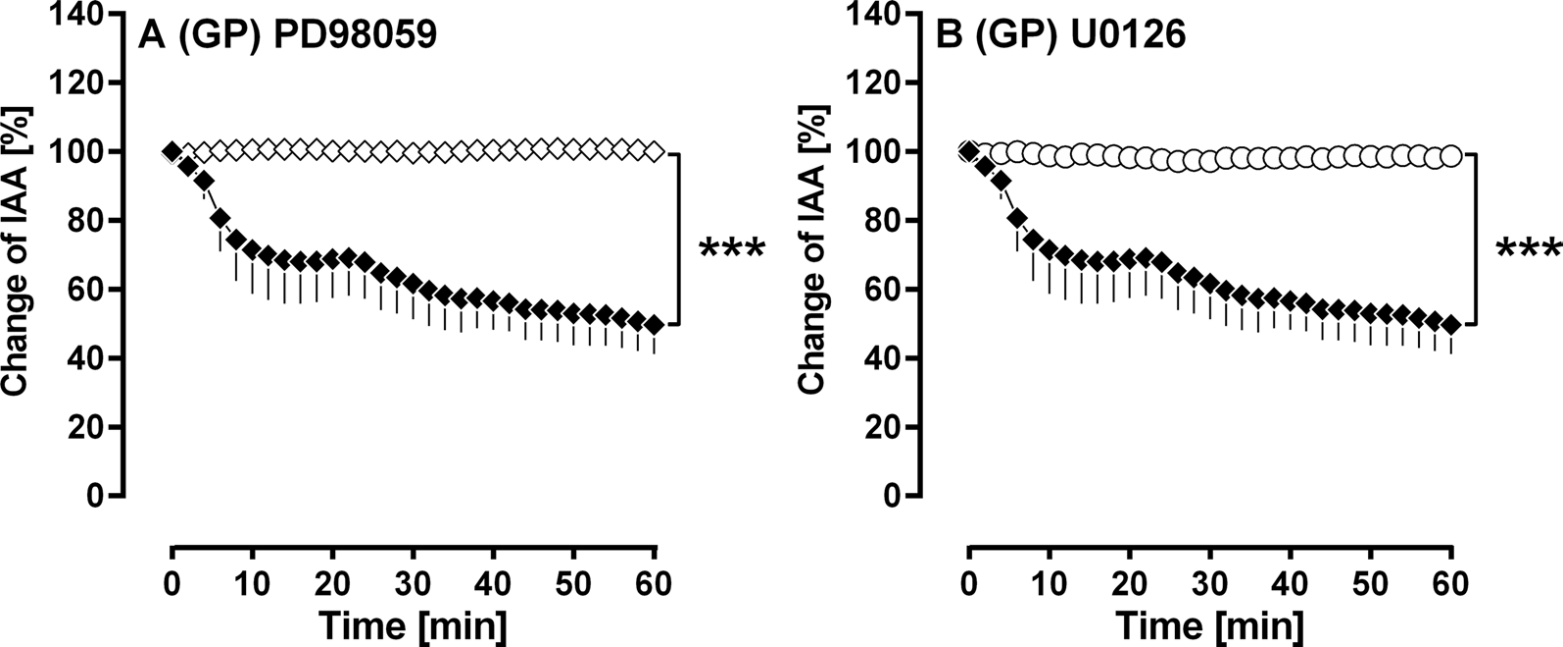
(GPs’ PCLS): Activation of MAP2K-signalling in PDGF-BB-induced bronchoconstriction. **A** Inhibition of MAP2K by PD98059 (◆) no pre-treatment/100 nM PDGF-BB (n = 4); (◇) pre-treatment with 50 µM PD98059/100 nM PDGF-BB (n = 4). **B** Inhibition of MAP2K by U0126 (◆) no pre-treatment/100 nM PDGF-BB (n = 4); (○) pre-treatment with 5 µM U0126/100 nM PDGF-BB (n = 4). **A/B** Statistics was performed by a LMM. p < 0.05 are considered as significant: *p < 0.05, ^**^p < 0.01 and ***p < 0.001

**Fig. 5:**
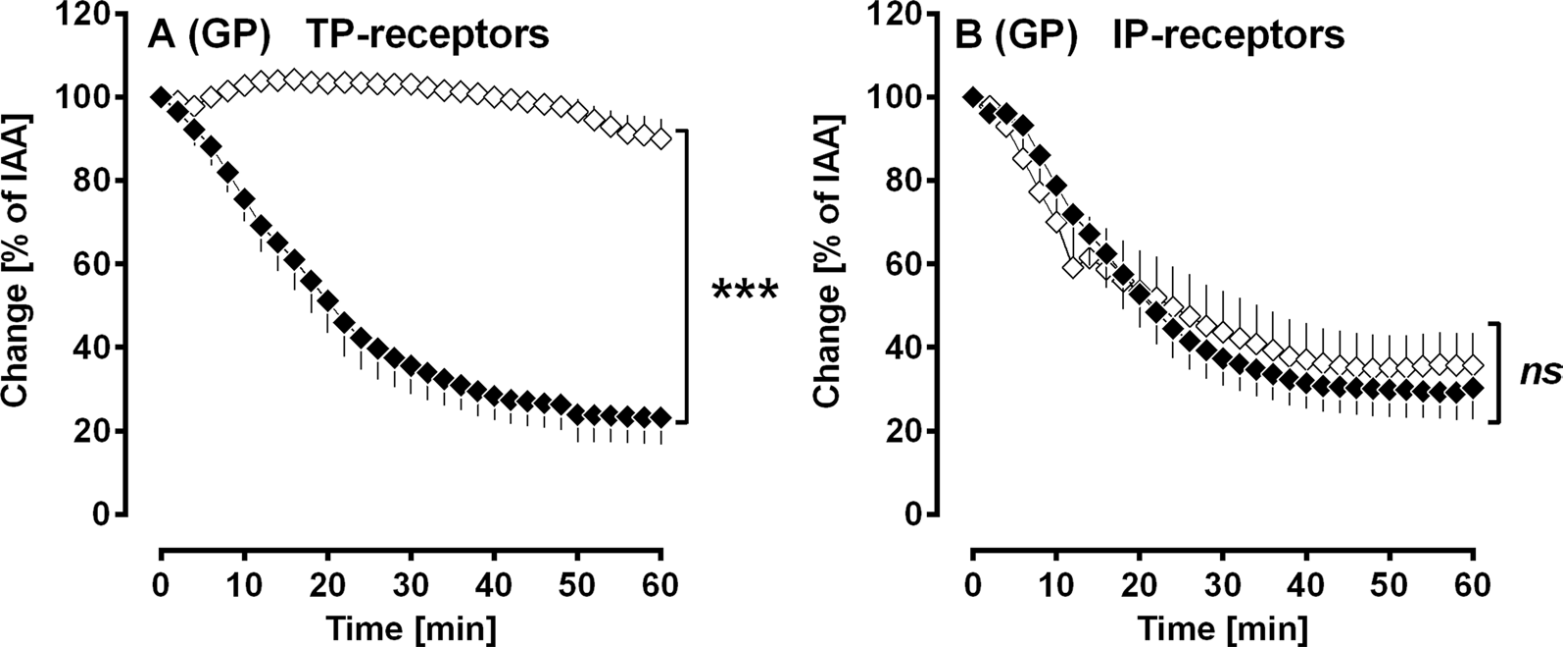
(GPs’ PCLS): Activation of TP- and IP-receptors in PDGF-BB-induced bronchoconstriction. **A** Inhibition of TP-receptors by SQ29548 (◆) no pre-treatment/100 nM PDGF-BB (n = 5); (◇) pre-treatment with 10 µM SQ29548/100 nM PDGF-BB (n = 5). **B** Inhibition of IP-receptors by RO-1138 (◆) no pre-treatment/100 nM PDGF-BB (n = 8); (◇) pre-treatment with 1 µM RO-1138/100 nM PDGF-BB (n = 8). **A/B** Statistics was performed by a LMM. p < 0.05 are considered as significant: *p < 0.05, **p < 0.01 and ***p < 0.001

Inhibition of MAP2K by 50 µM PD98059 (Fig. 4A) or 5 µM U0126 (Fig. 4B) completely prevented the contractile effect of PDGF-BB (p < 0.001).

#### Activation of TP- and IP-receptors in PDGF-BB-induced bronchoconstriction

Inhibition of TP-receptors with 10 µM SQ29548 (Fig. 5A) prevented PDGF-BB-induced contraction, whereas inhibition of IP-receptors with 1 µM RO-1138 (Fig. 5B) had no effect on PDGF-BB-induced bronchoconstriction.

#### No role of EP_1–4_-receptors in PDGF-BB-induced bronchoconstriction

**Fig. 6:**
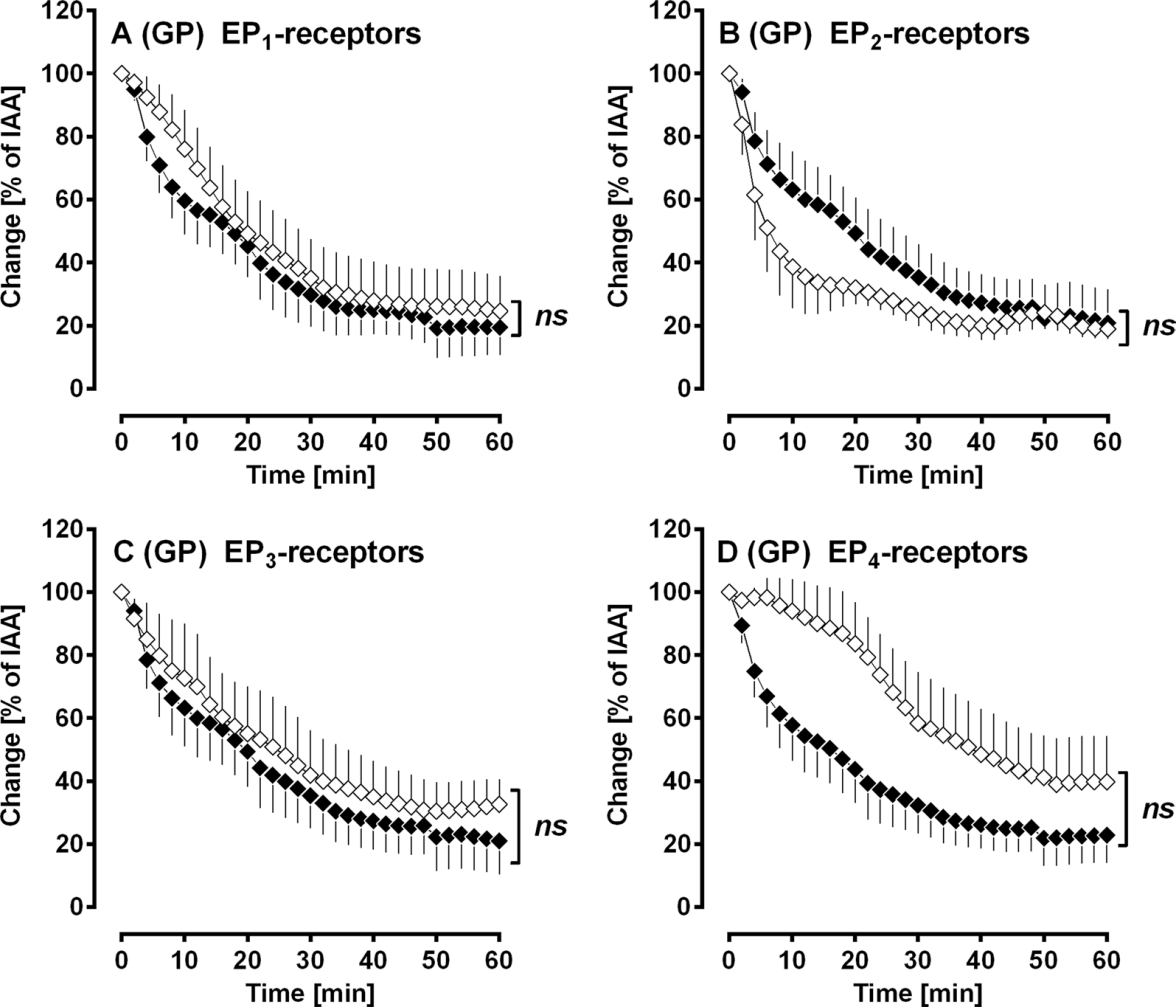
(GPs’ PCLS): No role of EP_1–4_-receptors in PDGF-BB-induced bronchoconstriction. **A** Inhibition of EP_1_-receptors by SC51322: (◆) no pre-treatment/100 nM PDGF-BB (n = 6); (◇) pre-treatment with 1 µM SC513222/100 nM PDGF-BB (n = 6). **B** Inhibition of EP_2_-receptors by PF0441 (◆) no pre-treatment/100 nM PDGF-BB (n = 5); (◇) pre-treatment with 1 µM PF0441/100 nM PDGF-BB (n = 5). **C** Inhibition of EP_3_-receptors by L798106: (◆) no pre-treatment/100 nM PDGF-BB (n = 5); (◇) pre-treatment with 1 µM L798106/100 nM PDGF-BB (n = 5). **D** Inhibition of EP_4_-receptors by L161982 (◆) no pre-treatment/100 nM PDGF-BB (n = 6); (◇) pre-treatment with 1 µM L161982/100 nM PDGF-BB (n = 6). **A**–**D** Statistics was performed by a LMM. p < 0.05 are considered as significant: *p < 0.05, **p < 0.01 and ***p < 0.001

Neither inhibition of EP_1_-receptors with 1 µM SC51322 (Fig. 6A), nor inhibition of EP_2_-receptors with 1 µM PF0441 (Fig. 6B), nor inhibition of EP_3_-receptors with 1 µM L796106 (Fig. 6C) influenced PDGF-BB-induced contraction. In contrast, inhibition of EP_4_-receptors with 1 µM L161982 (Fig. 6D) appears to lower the contractile effect of PDGF-BB; however, this effect was without statistical significance.

#### The role of actin polymerisation within PDGF-BB-induced bronchoconstriction

**Fig. 7:**
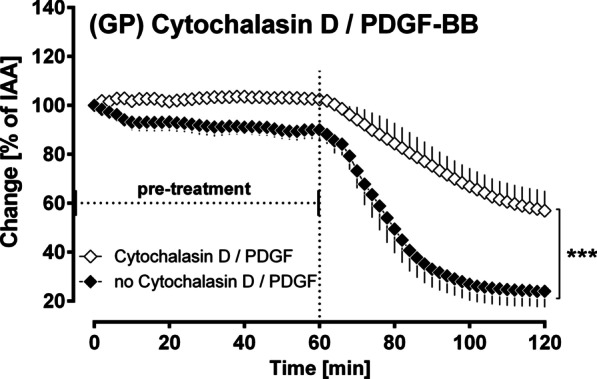
(GPs’ PCLS): The role of actin polymerisation within PDGF-BB-induced bronchoconstriction. Inhibition of actin polymerisation by cytochalasin D: (◆) no pre-treatment/100 nM PDGF-BB (n = 7); (◇) pre-treatment with 10 µM cytochalasin D/100 nM PDGF-BB (n = 7). Statistics was performed by a LMM. p < 0.05 are considered as significant: *p < 0.05, **p < 0.01 and ***p < 0.001

The control airways slightly contracted over the pre-treatment period of 60 min, whereas airways pre-treated with cytochalasin D neither contracted nor relaxed and the airway tone remained stable. After treatment with PDGF-BB, control airways strongly contracted to 23% of IAA (Fig. 4). In contrast, the airways which passed through inhibition of actin polymerisation by cytochalasin D, contracted only to 56% of IAA (p < 0.001; Fig. 7).

#### The role of Ca^2+^ and Ca^2+^-sensitisation within PDGF-BB-induced bronchoconstriction

**Fig. 8:**
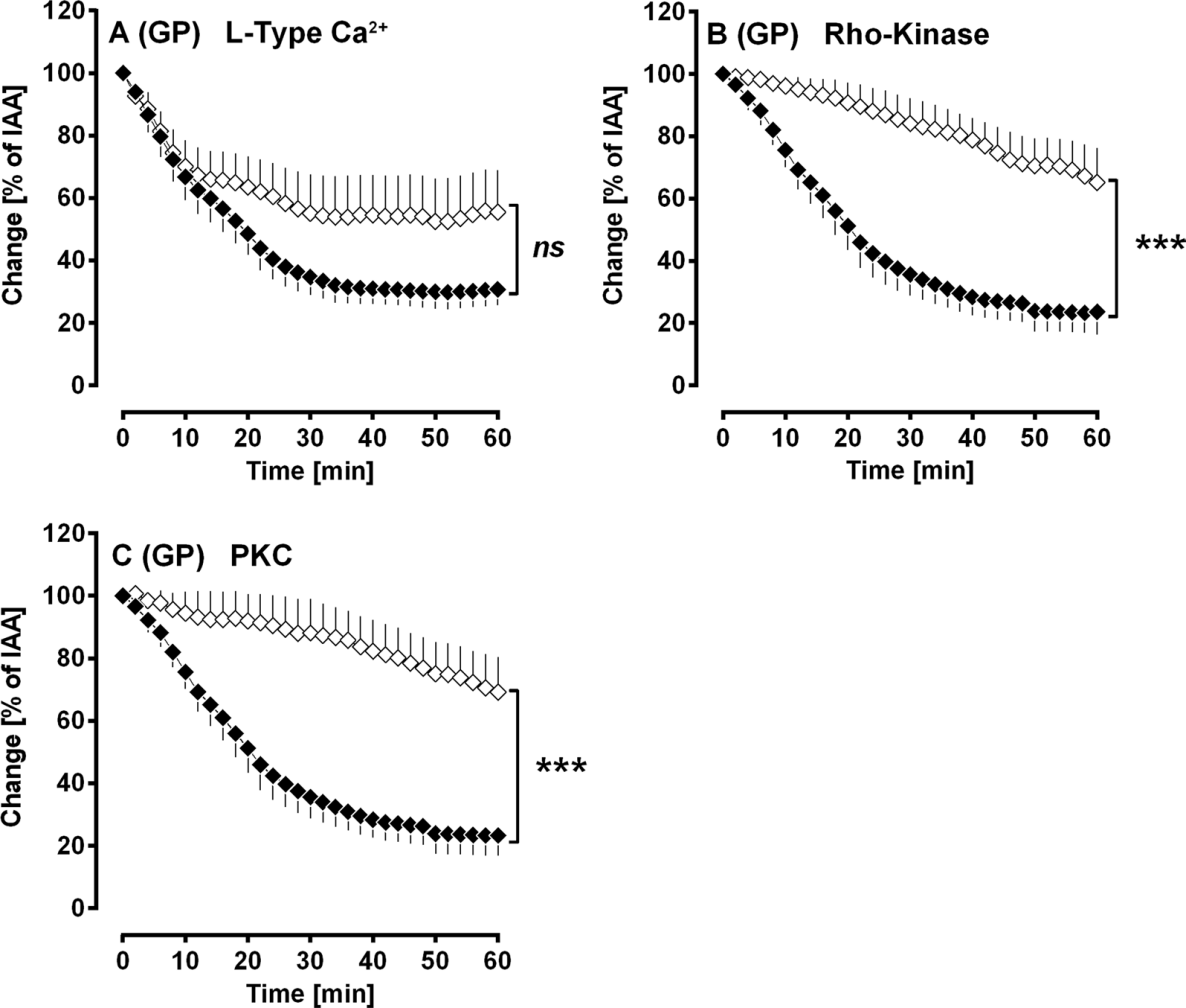
(GPs’ PCLS): The role of Ca^2+^ and Ca^2+^-sensitisation within PDGF-BB-induced bronchoconstriction. **A** Inhibition of voltage-gated Ca^2+^-channels by amlodipine: (◆) no pre-treatment/100 nM PDGF-BB (n = 7); (◇) pre-treatment with 100 nM amlodipine/100 nM PDGF-BB (n = 7). **B** Inhibition of Rho-Kinase by fasudile: (◆) no pre-treatment/100 nM PDGF-BB (n = 5); (◇) pre-treatment with 10 µM fasudile/100 nM PDGF-BB (n = 5). **C** Inhibition of PKC by calphostin C: (◆) no pre-treatment/100 nM PDGF-BB (n = 5); (◇) pre-treatment with 5 µM calphostin C/100 nM PDGF-BB (n = 5). **A**–**C** Statistics was performed by a LMM. p < 0.05 are considered as significant: *p < 0.05, **p < 0.01 and ***p < 0.001

Pre-treatment with the Ca^2+^-channel blocker amlodipine (100 nM) did not significantly influence PDGF-BB-induced contraction (Fig. 8A). In contrast, inhibition of Rho-Kinase by fasudile (10 µM) significantly reduced the contractile effect of PDGF-BB (Fig. 8B), as does inhibition of PKC by calphostin C (5 µM) (Fig. 8C).

## Discussion

PDGF and PDGFR play a critical role within the remodelling in chronic airway diseases [1, 49]. Additionally, TKIs are increasingly focused as possible therapeutic agents [3–7, 50]. In contrast to these considerations, the acute effects of PDGF-BB and TKIs on the airway tone, e.g. constriction or relaxation have not yet been studied intensively. Here, we show that PDGF-BB contracts airways of GPs and humans via activation of PDGFR. Vice versa, PDGF-BB-induced airway contraction was prevented by TKIs. Further, the TKI imatinib even relaxed ET-1 pre-constricted human airways.

### Effect of PDGF-BB on airway parameters

In the IPL (GP), PDGF-BB significantly reduced the tidal volume (Fig. 2A) and the dynamic compliance (Fig. 2B) of the lung. In addition, resistance tends to increase, however without statistical significance (Fig. 2C). The appearance of this bronchoconstrictive effect of PDGF-BB is supported by our results from GPs’ PCLS, where PDGF-BB also exerted distinct bronchoconstriction (Fig. 3A). In both experimental models, PDGF-BB-induced bronchoconstriction was completely prevented by TKIs. For example, in IPLs (Fig. 2A–C) pre-treatment with the PDGFR-α/β inhibitor imatinib completely avoided PDGF-BB-induced bronchoconstriction. Further in GPs’ PCLS, inhibition of PDGFR-β by SU6668 prevented PDGF-BB-induced bronchoconstriction (Fig. 3A), whereas inhibition of PDGFR-α by ponatinib had no effect (Fig. 3A), suggesting a dominant role of PDGFR-β within the contractile effect of PDGF-BB. The relevance of our findings is reinforced by the fact that PDGF-BB strongly contracted human airways (Fig. 3B) in PCLS, which was prevented by the PDGFR-α/β inhibitor imatinib (Fig. 3B). Further, imatinib also relaxed human airways after pre-constriction with ET-1, suggesting a relevant role of PDGFR within the regulation of the airway tone (Fig. 3C). This observation might be useful for approaches in asthma therapy.

Research in this field is scarce. Solely, Schaafsma et al. [51] demonstrated the bronchoconstrictive effect of PDGF-BB in GP tracheal strips, whereas Zhou et al. [10] proved the role of PDGFR within bronchoconstriction in rats lung slices.

### Mechanisms for PDGF-BB-induced bronchoconstriction

#### The role of MAP2K-signalling in PDGF-BB-induced bronchoconstriction

Our results indicate that MAP2K-signalling is of pivotal role within the bronchoconstrictive effect of PDGF-BB, as inhibition of MAP2K-signalling by PD98059 and U0126 (Fig. 4A, B) completely prevented PDGF-BB-induced bronchoconstriction. Accordingly, Schaafsma et al. [51] proved the contractile effect of PDGF-BB in the trachea of GPs, just as the involvement of MAP2K. In addition, PDGF-BB-induced contraction of pulmonary veins also depends on MAP2K-signalling [19]. These findings are in line with the role of TP-receptors within the contractile effect of PDGF-BB in GPs’ airways (Fig. 5A) and pulmonary veins [19]. Further, they are explained by the fact that PDGF-BB induces the activation of MAP2K, which itself stimulates phospholipase A_2_ (PLA_2_) and subsequent prostaglandin synthesis [52–55]. Consequently, MAP2K-signalling is highly involved in PDGF-BB-induced prostaglandin synthesis.

#### The role of prostanoids in PDGF-BB-induced bronchoconstriction

TP-receptors are highly involved within PDGF-BB-induced bronchoconstriction, as inhibition of TP-receptors by SQ29548 completely prevented the contractile effect of PDGF-BB (Fig. 5A). TP-receptors are primarily linked to G_αq/11_ and G_α12/13_. Induction of G_αq/11_ leads to the formation of IP_3_ and—by the release of Ca^2+^ from the sarcoplasmic reticulum—to increased intracellular Ca^2+^ levels [56, 57]; whereas activation of G_α12/13_ mediates the induction of RhoA/ROCK which itself inhibits the myosin light chain phosphatase. Finally, both signalling pathways provoke sustained contraction [58].

In contrast to the role of TP-receptors, PDGF-BB-induced activation of IP-receptors (Fig. 5B) appears to be without relevance, as inhibition of IP-receptors by RO-1138 did not alter the contractile effect of PDGF-BB. Additionally, activation of EP_1–4_-receptors by PDGF-BB (Fig. 6A–D) seems to be at most of minor impact. Our results are partly in contrast to those from Schaafsma et al. [51] who found that PDGF-BB-induced bronchoconstriction (GPs) depends on the activation of EP_1_-receptors [51]. Further, Zhou et al. [10] showed that PDGFR-downstream signalling involves the activation of EP_3_-receptors. Our present results are further in contrast to a former study in pulmonary veins of GPs. There, PDGF-BB-induced contraction of SMCs was shown to depend on the activation of TP-receptors, as well as on the activation of EP_1/3/4_-receptors [19].

Comparing between the findings of our present work on GP airways and a former work on GP pulmonary veins [19], it is evident that TP-receptors play a pivotal role within PDGF-BB-induced contraction. This is also supported by the fact that thromboxane B_2_ (TXB_2_), the inactive metabolite of thromboxane A_2_ (TXA_2_), is strongly increased in the perfusate of IPLs after treatment with PDGF-BB. This effect is prevented if IPLs were pre-treated with imatinib [19].

#### The role of actin polymerisation within PDGF-BB-induced bronchoconstriction

**Fig. 9:**
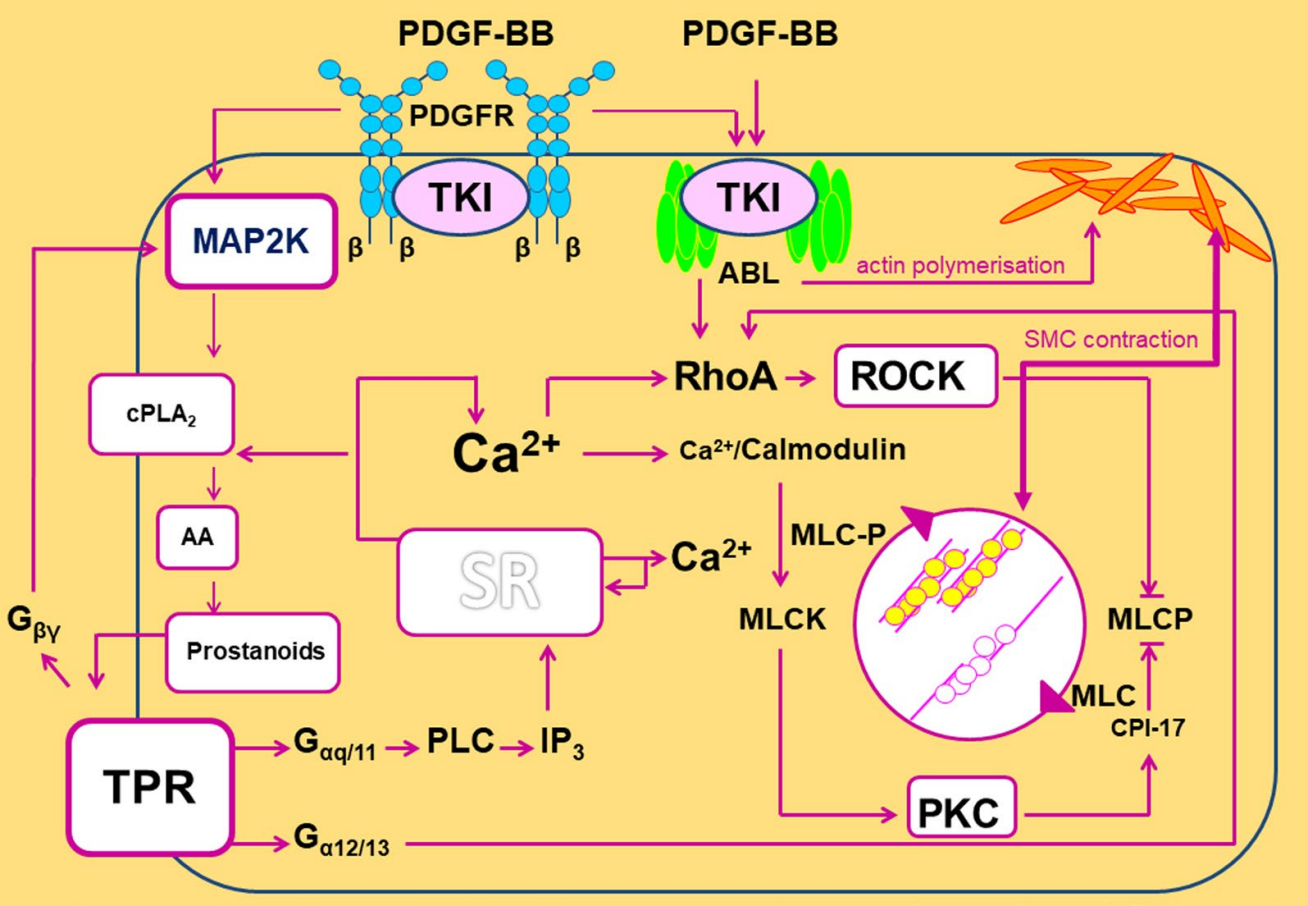
Mechanisms for PDGF-BB-induced bronchoconstriction. The contractile effect of PDGF-BB depends on the activation TP-receptors which are mainly coupled to G_α12/13_ [56, 57] activating Rho/ROCK and inhibiting thereby MLCP [58]. Further, TP-receptors (TPR) are coupled to G_αq/11_, leading to the formation of IP_3_ and to the release of calcium from the sarcoplasmic reticulum (SR) [56, 57]. Increased cytosolic calcium levels are leading to the activation of PKC which itself inhibits the myosin light chain phosphatase (MLCP), hence the actomyosin system remains activated and contraction of SMCs is intensified [58]. In addition, increased cytosolic calcium levels provoke the activation of Rho/ROCK [58, 67]. Last, TP-receptors (TPR) are linked to G_βγ_ activating MAPK-signalling. MAPK-signalling supports the activation of TP-receptors, as it strengthens the activation of the cytosolic PLA_2_ [52, 53], which leads to the formation of arachidonic acid (AA), serving as a substrate for the production of TXA_2_ [77]. Moreover, both PDGF-BB and PDGFR stimulate the abelson tyrosine kinase (Abl) [60, 61, 78] which acts downstream on Rho/ROCK [63, 64]. In contrast, the TKI imatinib, but not SU6668, inhibits Abl [62]. The stimulation of Abl by PDGF-BB and PDGFR is important for SMCs’ contraction, as Abl supports the polymerisation of subcortical actin filaments [59] strengthening the membrane for the transmission of the force generated by the actomyosin system. Hence, the stabilization of the cytoskeleton and the crossbridge cycling reinforce each other, leading to enhanced contraction [59]

Actin polymerisation is an important process within the regulation of the tone of smooth muscle cells (SMC) [59]. In this context, activation of PDGFR-β contributes—via the stimulation of SRC and abelson tyrosine kinase (Abl) (Fig. 9)—to actin polymerisation [60, 61]. Conversely, inhibition of PDGFR-β, e.g. by SU6668 or imatinib prevents this process. In addition, imatinib acts as a direct inhibitor of Abl [62]. Consecutively, imatinib prevents Abl downstream signalling such as actin polymerisation [62] and activation of RhoA/ROCK [63, 64]. Our data indicate that actin polymerisation is involved within PDGF-BB-induced bronchoconstriction, as its inhibition by cytochalasin D reduced the contractile effect of PDGF-BB (Fig. 7). Our results are supported by the facts that (1) actin polymerisation is of impact for the contractile effect of PDGF-BB in pulmonary veins (GP) [19] and (2) that actin polymerisation is of relevance for airway hyperresponsiveness [62, 65]. Further, Nayak et al. [66] recently demonstrated the additive effects of beta-agonists and Abl-inhibitors on the airway tone in murine PCLS and in mice with the flexiVent system [66].

#### Ca^2+^- release and Ca^2+^-sensitisation within PDGF-BB-induced bronchoconstriction

Studying the mechanisms of PDGF-BB-induced bronchoconstriction in GP PCLS, we found that inhibition of L-Type Ca^2+^ channels by amlodipine seems to play an at best minor role within the contractile effect of PDGF-BB (Fig. 8A). In contrast, inhibition of Rho-Kinase by fasudile (Fig. 8B) as well as inhibition of PKC by calphostin C (Fig. 8C) significantly reduced the contractile effect of PDGF-BB, suggesting a relevant role of Ca^2+^-sensitisation within the bronchoconstrictive effect of PDGF-BB. Our results are conflicting as Ca^2+^-sensitisation depends in part also on the increase of intracellular Ca^2+^ (Fig. 9) [58, 67]; e.g. Rho kinase mediated contraction of SMCs can be activated by Ca^2+^-independent and Ca^2+^-dependent mechanisms (Fig. 9), however activation of PKC is absolutely coupled on the release of Ca^2+^. Consecutively, Ca^2+^ should be involved within PDGF-BB-induced bronchoconstriction anyhow.

Our results are in contrast to those of Zhou et al. [10], who showed in rat lung slices that PDGF-BB-associated airway constriction depends on increased Ca^2+^-levels. The diverging results might be explainable by the different species, GPs versus rats. Yet, this aspect should not be the only reason for a possible discrepant regulation of the tone of SMCs. In fact, in contrast to our present results, L-Type Ca^2+^-channels play a dominant role within PDGF-BB-induced constriction in pulmonary veins of GPs, whereas Ca^2+^-sensitisation does not [19].

### The bronchorelaxant effect of Imatinib

Beyond the contractile effects of PDGF-BB, PDGFR-inhibition by imatinib exerts bronchorelaxation in ET-1-pre-constricted human airways. In contrast, if airways were not pre-constricted, imatinib did not mediate relaxation, but it prevented PDGF-BB-induced contraction. Our results are supported by those of Chopra et al. [50] who proved a relaxant effect of the TKIs ST638, genistein and tyrphostin A47 in isolated bronchioles of rats. Recently, Nayak et al. [66] showed in murine PCLS and in a murine in vivo model (flexiVent) that TKIs and β-agonist act synergistic within the context of bronchorelaxation. Beyond, the relaxant effects of several TKIs, e.g. nilotinib, imatinib and nintedanib (unpublished data) have been proven in different tissue of various species [17, 22, 68–70].

Beyond the Abl-inhibiting properties of imatinib [62], it should be considered that imatinib relaxed human airways pre-constricted with ET-1. Within this context it is relevant that ET-1 downstream signalling [71–74] involves the release of TXA_2_ and subsequently the activation of TP-receptors, as a common pathway of ET-1-induced contraction. Furthermore, it is possible that imatinib interacts directly with TP-receptors. Since long, TP-receptors are focused as possible targets in the therapy of chronic asthma [75, 76]. Imatinib might contribute to generate new therapeutic approaches in asthma. Within this context inhaled imatinib may enlarge the existing repertoire of topical application.

In conclusion, (1) PDGF-BB contracts airways. (2) Imatinib (perfused/nebulised) prevents the contractile effects of PDGF-BB. (3) Imatinib relaxes ET-1 pre-constricted human airways. Finally, PDGFR might act as a central platform in chronic airway disease, e.g. IPF or asthma. Within this context, MAP2K-signalling, activation of TP-receptors, actin polymerisation and Ca^2+^-sensitisation appear to play a central role. Thus, TKI-inhibition might be a beneficial and prospective strategy in asthma and airway hyperresponsiveness.

## Data Availability

The datasets generated and analysed during the current study are available from the corresponding author on reasonable request.

## Abbreviations

Abl: Abelson tyrosine kinase
Cdyn: Dynamic compliance
EP-receptor: Prostaglandin E_1–4_ receptor
ET-1: Endothelin-1
GPs: Guinea pigs
IAA: Initial airway area
IPF: Idiopathic pulmonary fibrosis
IPL: Isolated perfused lung
IP-receptor: Prostacyclin receptor
LMM: Linear mixed model analyses
PCLS: Precision-cut lung slices
PDGF: Platelet derived growth factor
PDGFR: PDGF-receptor
PKC: Protein kinase C
P_LA_: Left atrial pressure
P_PA_: Pulmonal arterial pressure
Res: Resistance
SMC: Smooth muscle cell
TKIs: Tyrosine kinase inhibitors
TP-receptor: Thromboxane prostanoid receptor
TV: Tidal volume
TXB_2_: Thromboxane B_2_, inactive metabolite of thromboxane A_2_
TXA_2_: Thromboxane A_2_
